# A Systems Biology Approach Identifies a R2R3 MYB Gene Subfamily with Distinct and Overlapping Functions in Regulation of Aliphatic Glucosinolates

**DOI:** 10.1371/journal.pone.0001322

**Published:** 2007-12-19

**Authors:** Ida Elken Sønderby, Bjarne Gram Hansen, Nanna Bjarnholt, Carla Ticconi, Barbara Ann Halkier, Daniel J. Kliebenstein

**Affiliations:** 1 Plant Biochemistry Laboratory, Department of Plant Biology, Center for Molecular Plant Physiology (PlaCe), Faculty of Life Sciences, University of Copenhagen, Copenhagen, Denmark; 2 Department of Plant Sciences, University of California at Davis, Davis, California, United States of America; Purdue University, United States of America

## Abstract

**Background:**

Glucosinolates are natural metabolites in the order Brassicales that defend plants against both herbivores and pathogens and can attract specialized insects. Knowledge about the genes controlling glucosinolate regulation is limited. Here, we identify three R2R3 MYB transcription factors regulating aliphatic glucosinolate biosynthesis in *Arabidopsis* by combining several systems biology tools.

**Methodology/Principal Findings:**

*MYB28* was identified as a candidate regulator of aliphatic glucosinolates based on its co-localization within a genomic region controlling variation both in aliphatic glucosinolate content (metabolite QTL) and in transcript level for genes involved in the biosynthesis of aliphatic glucosinolates (expression QTL), as well as its co-expression with genes in aliphatic glucosinolate biosynthesis. A phylogenetic analysis with the R2R3 motif of *MYB28* showed that it and two homologues, *MYB29* and *MYB76*, were members of an *Arabidopsis*-specific clade that included three characterized regulators of indole glucosinolates. Over-expression of the individual *MYB* genes showed that they all had the capacity to increase the production of aliphatic glucosinolates in leaves and seeds and induce gene expression of aliphatic biosynthetic genes within leaves. Analysis of leaves and seeds of single knockout mutants showed that mutants of *MYB29* and *MYB76* have reductions in only short-chained aliphatic glucosinolates whereas a mutant in *MYB28* has reductions in both short- and long-chained aliphatic glucosinolates. Furthermore, analysis of a double knockout in *MYB28* and *MYB29* identified an emergent property of the system since the absence of aliphatic glucosinolates in these plants could not be predicted by the chemotype of the single knockouts.

**Conclusions/Significance:**

It seems that these cruciferous-specific *MYB* regulatory genes have evolved both overlapping and specific regulatory capacities. This provides a unique system within which to study the evolution of *MYB* regulatory factors and their downstream targets.

## Introduction

Glucosinolates are amino acid-derived natural plant products characteristic of the order Brassicales, including the agriculturally important oilseed rape, the Brassica vegetables and the model plant *Arabidopsis* [1], [2]. Glucosinolates are major defensive metabolites against both herbivores and pathogens and function as attractants for specialized insects [2], [3]. In addition, glucosinolates have gained increasing importance due to their function as flavour compounds and cancer-preventive agents in the human diet, as well as their potential for use as biopesticides [4].

**Figure 1:**
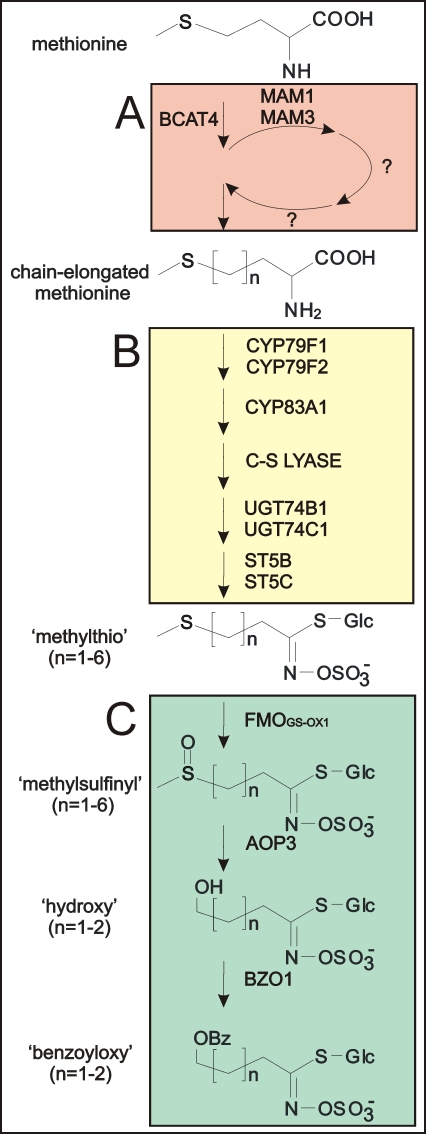
Biosynthetic pathway of aliphatic glucosinolates in *Arabidopsis* Col-0. A. The chain-elongation machinery. Methionine enters the chain-elongation cycle via deamination by BCAT4 and is subsequently condensed with acetyl-CoA in a reaction catalyzed by MAM1 and MAM3. MAM1 can catalyze one to four condensation cycles, whereas MAM3 catalyzes one to six cycles. Subsequently, an isomerization and oxidation-decarboxylation step occurs and the molecule can re-enter the cycle or enter the core pathway following a transamination step. B. Synthesis of the core methylthio glucosinolate structure. The first enzymatic step has side chain specificity with CYP79F1 that converts both short- and long- chained methionine derivatives (n = 1–6) to the corresponding aldoxime whereas CYP79F2 only takes the long-chained methionine derivatives (n = 5–6). C. Secondary modifications. Short- and long-chained methylthio glucosinolates can be secondarily modified to methylsulfinyl glucosinolates in Col-0 leaves. In the seeds, the short-chained methylsulfinyl glucosinolates can be further modified to hydroxy and benzoyloxy forms. Characterized enzymes in the pathway are noted next to the reaction arrows.

Glucosinolates are divided into aliphatic, aromatic and indole glucosinolates dependent on the nature of the amino acid from which they are derived. The three subgroups have a pathway for the synthesis of the core structure, where distinct, but homologous genes catalyze the conversion of amino acids to the corresponding glucosinolate. Methionine-derived glucosinolates are modified via elongation of the side chain prior to entering the pathway for the synthesis of the core glucosinolate structure. Short-chained, aliphatic glucosinolates have been through one to four cycles of the chain-elongation machinery, whereas the long-chained have been through five or six cycles (Figure 1). Furthermore, glucosinolate structures are often secondarily modified on their side chains with e.g. hydroxylations and alkenylations. The completion of the *Arabidopsis* genomic sequence has resulted in identification of most of the biosynthetic genes involved in the formation of the core structure, the chain-elongation machinery and secondary modifications (Figure 1).

Our knowledge about the genes controlling glucosinolate regulation is limited to a few regulatory genes. This is in contrast to the complex regulation known for glucosinolate content which includes herbivore, pathogen, biotic and abiotic stress responses [5]–[7]. Two genes are known to specifically regulate indole glucosinolates. *ATR1*/*MYB34* was identified as a gain-of-function mutant, *atr1D,* in a screen for mutants with **a**ltered **t**ryptophan **r**egulation [8], [9]. The transcript levels of *CYP79B2, CYP79B3* and *CYP83B1,* which encodes for enzymes in the biosynthesis of indole glucosinolates, are up- and down-regulated in *atr1D* and a knockout mutant, respectively [9]. *MYB51/HIG1*, a close homologue of *ATR1*/*MYB34*, was identified as an activation tagged line with high indole glucosinolates [10]. Complementation studies suggest that *MYB34* and *MYB51* have overlapping, but distinct functions in regulating biosynthetic genes for indole glucosinolates.

Other regulators have been shown to regulate both indole and aliphatic glucosinolates. *TFL2*, a plant homologue of the animal heterochromation protein (HP1) was shown to affect both aliphatic and indole glucosinolates, probably through indirect developmental effects [11], [12]. A screen for high-glucosinolate mutants identified *IQD1*, a calmodulin-binding nuclear protein, as a potential regulator [13]. The *iqd1*mutant has elevated levels of both aliphatic and indole glucosinolates, although transcript levels of genes in the indole glucosinolate pathway are up-regulated and transcripts in the aliphatic pathway are repressed [13]. Over-expression of a third transcription factor, *AtDof1.1*, results in an approximately two-fold increase in the levels of both indole and short-chained aliphatic glucosinolates. Accordingly, these *35S:Dof1.1* lines up-regulate transcript levels for *ATR1/MYB34* and the genes involved in indole glucosinolate biosynthesis, *CYP79B2, CYP79B3* and *CYP83B1,* as well as of *MAM1* involved in chain-elongation machinery for aliphatic glucosinolates [14]. Although these three regulatory genes, *TFL2, IQD1* and *AtDof1.1*, affect both aliphatic and indole glucosinolates, many studies indicate that aliphatic and indole glucosinolates are typically not co-ordinately regulated in response to pathogens, hormones or insect herbivores [5]–[7], [15].

When we initiated our study, the aim was to identify regulators specific for biosynthesis of the aliphatic glucosinolates, which had not yet been identified. Our approach was to exploit the natural variation in production of glucosinolates among *Arabidopsis* ecotypes using QTL mapping to identify candidate regulators. We have used a combination of metabolite QTL and expression QTL (eQTL) analysis within the Bay-0×Sha Recombinant Inbred Lines (RILs) to show that a region of the genome controls variation in both the accumulation of aliphatic glucosinolates and transcript levels for the aliphatic biosynthetic genes. Combining this data with information from co-expression databases and phylogenetic analysis identified three candidate transcription factors for aliphatic glucosinolates, *MYB28, MYB29*, and *MYB76*, belonging to an *Arabidopsis* specific MYB group. Glucosinolate and transcript profiling of over-expression lines indicated that *MYB28*, *MYB29* and *MYB76* positively regulate accumulation of aliphatic glucosinolates and the corresponding transcripts. This was further supported by a decrease in levels of aliphatic glucosinolates in T-DNA insertion mutants of *MYB28*, *MYB29* and *MYB76*, where the mutants reveal differential specificity for chain lengths. Further, a double knockout mutant in *MYB28* and *MYB29* was devoid of aliphatic glucosinolates and displayed very low levels of transcripts not predicted from the single knockout mutants. During our study, Hirai *et al.* (2007) provided evidence that *MYB28* is a regulator of aliphatic glucosinolates using an omics-based approach and Gigolashvili *et al.* (2007) has used activation tag approach to identify *MYB28* as regulator of aliphatic glucosinolates. Their data is discussed in relation to our data showing that all three MYBs are regulators of aliphatic glucosinolates independent of biotic stresses.

## Results

### A QTL on Chromosome V Appears to be a Regulatory QTL

**Figure 2:**
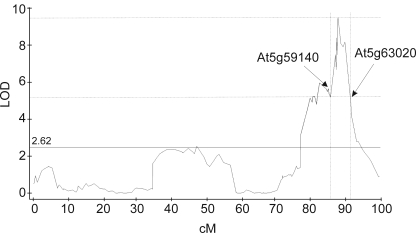
Bay-0×Sha QTL for Total Content of Aliphatic Glucosinolates on Chromosome V. The total content of aliphatic glucosinolates was measured in two independent experiments on 403 lines from the Bay-0×Sha RIL population. The LOD plot from QTL Cartographer CIM module for QTL on chromosome V is shown. The significance threshold was obtained by 1,000 random permutations within QTL Cartographer. A 4-LOD confidence interval around the QTL is depicted (vertical dotted lines) and the arrows show the position of the genes marking the 4 LOD interval.

Previous work using the Cvi-1×Col-0 and Cvi-1×L*er Arabidopsis* RIL populations identified a metabolite QTL controlling the accumulation of total aliphatic glucosinolates at the bottom of Chromosome V [5], [16], [17]. An analysis of the Bay-0×Sha RILs for total content of aliphatic glucosinolates showed the presence of a similar QTL within this population (Figure 2).

Similar to QTLs controlling metabolite accumulation, regions in the genome that control variation in the transcript level for a given gene (eQTL) can be identified [18]–[21]. Using microarray technology, genome-wide expression profiling of 211 individuals from the Bay-0×Sha RIL population was performed to map global eQTLs [22]. eQTLs can be separated into *cis-* and *trans-*acting eQTLs. The *cis*-eQTLs is likely caused by sequence variation around the region of the gene itself, e.g. a promoter polymorphism that gives rise to differential expression of the gene in the two parents. *trans*-eQTLs, on the other hand, are due to polymorphisms at another location on the genome than the actual physical position of the gene [23]. Such a region could potentially contain variation within a regulatory factor, potentially a transcription factor. Rather than looking for regions with *trans*-eQTLs for one gene, one can look for regions with *trans*-eQTLs for a set of genes (a gene network analysis) that share a common biological function, e.g. a metabolic pathway [24]. In this way, eQTL analysis may potentially identify master regulators of a network.

A previously conducted gene network analysis based on the genes of both the aliphatic and indole glucosinolate biosynthetic pathways suggested that the QTL on Chromosome V (Figure 2) may affect glucosinolate content via altered transcript levels for the aliphatic glucosinolate biosynthetic genes [24]. We utilized the transcript data set from 211 RILs from the Bay-0×Sha population [22] to test if the individual biosynthetic genes in the aliphatic pathway had eQTLs controlling variation in their transcript levels that also co-localized to the metabolite QTL on chromosome V. 15 of the 25 genes characterized or predicted to be involved in the biosynthesis or regulation of aliphatic glucosinolates had a statistically significant *trans-*eQTL that co-localized with the metabolite QTL at the bottom of chromosome V (Figure 2 and Table S1). These data suggest that variation in a regulatory factor within this region may control the metabolite QTL and eQTL for aliphatic glucosinolates. Supporting this idea is the observation that for 13 of the 15 significant genes, the Sha allele at this *trans-*eQTL led to higher transcript levels which correlates with the same Sha allele having elevated aliphatic glucosinolate content. Thus, this locus may enhance the total amount of aliphatic glucosinolates by increasing transcript level for some of the biosynthetic genes.

### The candidate regulator of aliphatic glucosinolates belongs to the R2R3 transcription factor family

To identify candidate regulatory genes within this QTL, we scanned all the genes physically located within the 4-LOD interval of the QTL for the presence of a *cis*-eQTL controlling their expression (Figure 2). Regulatory genes with a *cis*-eQTL in this 4-LOD interval have the potential to result in *trans*-eQTL for genes localized in other parts of the genome (Table S2). The 4-LOD interval corresponded to a region spanning approximately 400 genes from *At5g59140* to *At5g63020* (Figure 2). Among these 400 genes, we identified 63 genes whose difference in transcript levels between Bay-0 and Sha identified a *cis*-eQTL (Table S2).

This list of 63 candidate genes was next filtered to identify genes whose expression correlated with characterized genes of the aliphatic glucosinolate biosynthetic using the public co-expression database ATTED-II (http://www.atted.bio.titech.ac.jp/) (Table S2). The filtering step left only one candidate gene, *At5g61420*, that co-expressed with biosynthetic genes for aliphatic glucosinolates and that had a *cis*-eQTL which led to the Sha allele having approximately 3-fold more transcript than Bay. Further, this gene is physically located within 1 cM of the QTL peak as defined using both the metabolite and eQTL data with a dense marker map within this region (Figure 2) [25].

*At5g61420* is annotated as *MYB28*, a member of the R2R3 MYB transcription factor family. The R2R3 MYB family of transcription factors is plant-specific and holds ∼125 members in *Arabidopsis* [26]. It is characterized by having two repeats of DNA binding domains termed R2 and R3 in the N-terminal end, each containing three regularly spaced conserved tryptophan residues [26], while the C-terminal end is believed to confer DNA activation or repression. R2R3 MYB transcription factors are involved in many plant-specific processes such as the phenylpropanoid pathway [27], control of cell shape and differentiation [28], cold regulation [29], [30], stomatal aperture [31] as well as regulation of indole glucosinolates [9], [32].

**Figure 3:**
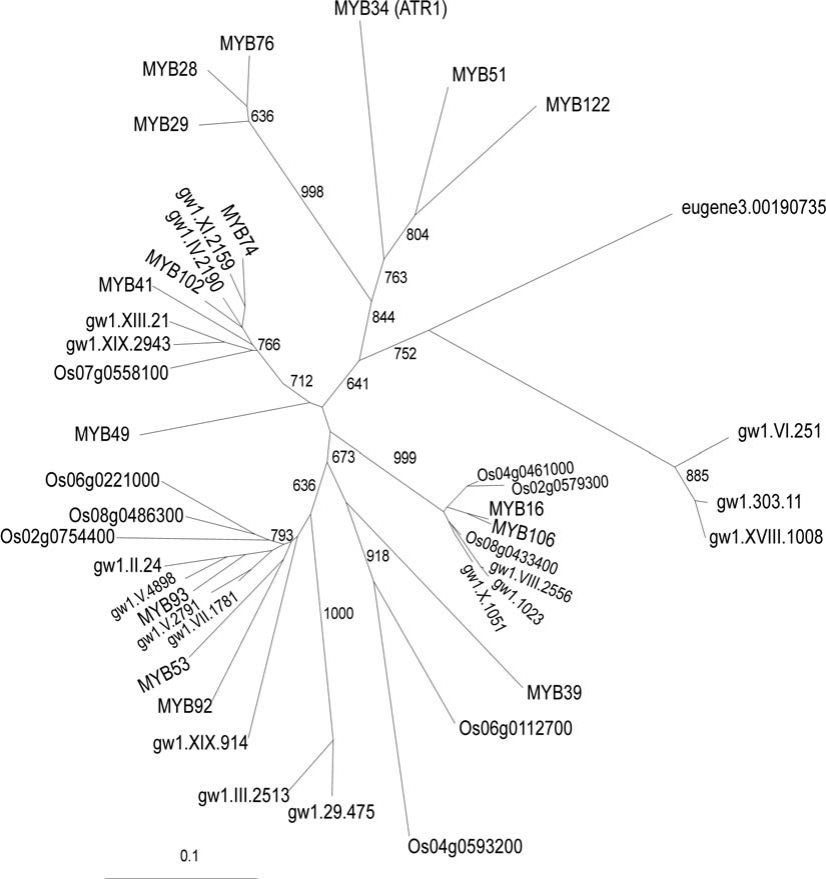
Phylogenetic tree of plant MYBs related to *MYB28*. The entire MYB complement from the genomic sequence for rice, *Arabidopsis* and poplar with a BLAST *P* value of 10e^−60^ or lower when tested against the *MYB28, MYB29, MYB76* or *ATR1/MYB34* R2R3 domain, were obtained and translated into their corresponding amino acid sequence. A complete neighbor joining tree with 1000 bootstraps was conducted in ClustalX and the unrooted cladogram is shown. All branches were supported by, at minimum, 630 bootstraps. A distance scale is shown to the bottom left. Table S3 contains the identification numbers for the sequences and Figure S1 the alignments.

Since glucosinolates are limited to the order Brassicales, we hypothesized that an aliphatic glucosinolate regulatory factor, similar to the *S*-oxygenating FMO of aliphatic glucosinolates [33], would not have homologues within the published rice and poplar genome sequences because both species do not contain glucosinolates. Using the *MYB28* R2R3 motif, we identified all related protein sequences in the genome of *Arabidopsis*, rice and poplar, and used the R2R3 domains from these proteins to create a phylogenetic tree (Figure 3, Figure S1, Table S3). The tree was similar using both neighbor joining and maximum parsimony analysis (data not shown). The unrooted tree shows that the closest homologues of *MYB28* are *MYB29*, *MYB76*, *MYB122*, *MYB51* and *MYB34*. The absence of annotated proteins from the dicot poplar and rice genomes within this clade suggests that the MYBs in this clade are specific to at least *Arabidopsis*. Additionally, no proteins that cluster within this clade were identified in sorghum, maize or any EST library. In support of the potential function of this MYB clade in glucosinolate production is the fact that *MYB51*, *MYB34* and *MYB122* have been shown to regulate indole glucosinolates levels in plants [9], [32]. Additionally, during the preparation of this manuscript, *MYB28* was shown to be a regulator of aliphatic glucosinolates [10], [34]. This generates the hypothesis that this clade arose to regulate glucosinolates and that the *MYB28/29/76* sub-clade may constitute regulators of aliphatic glucosinolates.

*MYB29(At5g07690)* and *MYB76(At5g07700)* co-localize with a QTL at the top of chromosome V for aliphatic glucosinolate content in two published QTL populations, Cvi-1×L*er* and Cvi-1×Col-0 [5], [16]. Analysis of gene expression data in the AtGenExpress database (http://www.weigelworld.org/resources/microarray/AtGenExpress/) and the Expression Level Polymorphism project database (http://elp.ucdavis.edu/database.htm) revealed that *MYB29* and *MYB76* are differentially expressed in Cvi and L*er* as well as in Cvi and Col-0. Further, *MYB29* and *MYB76* show co-expression with characterized and predicted aliphatic glucosinolate genes in the ATTED-II public database (Table S4). Given the phylogenetic relationship, association with multiple aliphatic glucosinolate QTLs and co-expression relationships, we proceeded to test the ability of all three MYBs, 28, 29 and 76, to regulate aliphatic glucosinolate accumulation and gene expression.

### Accumulation of aliphatic glucosinolates in 35S:MYB over-expression lines

Transgenic plants with the three candidate MYB genes driven by the Cauliflower Mosaic Virus 35S promoter were generated to test if the genes can regulate production of aliphatic glucosinolates in *Arabidopsis*. Ten *35S:MYB28, 35S:MYB29* and *35S:MYB76* lines were chosen for foliar glucosinolate analysis. Four, three and four lines of *35S:MYB28*, *35S:MYB29* and *35S:MYB76*, respectively, exhibited elevated levels of aliphatic glucosinolates. For further analysis, we chose two independent transgenic lines for each *35S:MYB* construct showing a significant increase in aliphatic glucosinolates.

Glucosinolate analyses on three-week-old rosette leaves showed that over-expression of all three *MYB* genes led to increased total content of aliphatic glucosinolates in comparison to wild-type Col-0 (Table 1 and 2, Table S5). In contrast, the level of total indole glucosinolates was not significantly affected (Table 1 and 2, Table S5). All plant experiments were conducted independently at two locations (University of Copenhagen and UC Davis) and the data combined into the presented ANOVA (Table 1 and 2, Table S5). The ANOVA showed that glucosinolate levels between the two selected transgenic lines for each *35S:MYB* transgene were not significantly different (Table S5). This allows us to combine the mean of the two independent lines to test for the effect of introducing *35S:MYB* transgenes into wildtype.

**Table 1:** Average Foliar Glucosinolate Content in plants containing the *35S:MYB28* transgene.

Trait	Col-0		*MYB28*		Sig
	N = 13		N = 32		
	Mean	SE	Mean	SE	
3MSP	41.3	26.8	44.6	9	
4MTB	48.7	9.2	23.8	3.6	**
4MSB	121.1	25.9	312.7	39.3	**
5MSP	12.3	7	36.5	3.3	**
8MTO	2.4	0.4	1.9	0.4	
8MSO	32.7	8.1	29.9	5.8	
Total Aliphatic	261	69.5	451.7	49.9	*
I3M	114.3	21.7	75	7.3	*
4MO-I3M	21.4	2.8	32.5	2.5	**
NMO-I3M	20.5	6.6	22.7	4.2	
Total Indole	156.2	28.6	130.1	10.1	

Mean shows the average glucosinolate content in pmol per mg of tissue using the abbreviations in Table S6. SE is the standard error of the mean for that line. This data represents two independent biological replicates using 13 plants for Col-0 and 32 plants containing the *35S:MYB28* transgene; 16 plants each from two independent transgenic lines. Sig indicates the *P* value of the difference between Col-0 and transgenic lines containing the *35S:MYB28* transgene as determined by ANOVA (Table S5). One asterisk represents a *P* value between 0.05 and 0.005, while two asterisks is a *P* value below 0.005. Cells with no asterisk represent non-significant *P* values, those greater than 0.05. N represents the total number of independent samples per genotypic class.

**Table 2 pone-0001322-t002:** Average Foliar Glucosinolate Content in plants containing the *35S:MYB29* or *35S:MYB76* transgene.

Trait	Col-0		*MYB29*			*MYB76*		
	N = 19		N = 31			N = 24		
	Mean	SE	Mean	SE	Sig	Mean	SE	Sig
3MSP	19	1.7	35.9	3.9	**	43.5	3.7	**
4MTB	42.9	6.5	37.6	5.8	**	28.6	5.7	**
4MSB	141.7	10	314.7	34.2	**	366.8	31.5	**
5MSP	5.9	0.4	14.1	1.9	**	16.2	2.3	**
8MTO	4.3	0.4	3.9	0.6		3.8	0.4	
8MSO	25.8	2.7	42.4	2.5	**	55.1	5.3	**
Total Aliphatic	250.4	15.7	458.7	38.1	**	528.3	41.2	**
I3M	52.3	6.6	63.6	5.5		65.4	9.2	
4MO-I3M	11.6	2.6	20.6	2.4		22	4	*
NMO-I3M	45.4	9.1	27.4	6.9		29.3	7.1	
Total Indole	109.3	8.3	111.6	9		116.7	11.5	

Mean shows the average glucosinolate content in pmol per mg of tissue using the abbreviations in Table S6. SE is the standard error of the mean for that line. This data represents two independent biological replicates using 19 plants for Col-0, 31 plants containing the *35S:MYB29* transgene (16 and 15 plants from two independent transgenic lines) and 24 plants containing the *35S:MYB76* transgene (twelve plants each from two independent transgenic lines). Sig indicates the *P* value of the difference between Col-0 and transgenic lines containing, respectively, the *35S:MYB29* and *35S:MYB76* transgene as determined by ANOVA (Table S5). One asterisk represents a *P* value between 0.05 and 0.005 while two asterisks is a *P* value below 0.005. Cells with no asterisk represent non-significant *P* values, those greater than 0.05. N represents the total number of independent samples per genotypic class.

In addition to affecting total content of aliphatic glucosinolates, the three MYB genes altered the composition of the aliphatic glucosinolates present in the leaves. All three *35S:MYB* transgenes resulted in significantly elevated levels of the short-chained 4MSB and 5MSP whereas the level of 4MTB was significantly lowered (Table 1 and 2, Table S5). A list of the glucosinolate abbreviations used is provided in Table S6. In contrast, the different *35S:MYB* transgenes exhibited divergent effects on long-chained aliphatic glucosinolates, e.g. methylsulfinyloctyl glucosinolate, 8MSO. Over-expression of *MYB29* and *MYB76* conferred a significant increase in 8MSO levels, whereas over-expression of *MYB28* did not alter the content of 8MSO (Tables 1 and 2). In a separate experiment, we tested for potential influences from maternal effects by selecting progeny segregating for the presence and absence of transgene from heterozygous T_2_ plant for each selected transgenic line. Previous analysis has shown that maternal effects can be a significant determinant of glucosinolate accumulation [35]. A comparison of segregants with the transgene to those without the transgene showed that in all instances, the presence of the *35S:MYB* transgene led to increased content of aliphatic glucosinolates, irrespective of the mother plant (data not shown).

Glucosinolate contents and profiles vary between different tissues [36], [37]. To investigate whether over-expression of the three MYB transcription factors conferred changes to the levels and composition of aliphatic glucosinolates in seeds as well, glucosinolates were extracted and analyzed from seeds originating from the plants used for glucosinolate analysis in leaves. All *35S:MYB28*, *35S:MYB29* and *35S:MYB76* lines showed elevated levels of aliphatic glucosinolates in seeds (Table 3, Table S7). Similar to what was observed in foliar tissue, the increase of aliphatic glucosinolates in seeds within the *35S:MYB28* lines was entirely due to a rise in short-chained aliphatic glucosinolates. In fact, a significant decrease in long-chained aliphatic glucosinolates was observed (Table 3). Seeds of *35S:MYB29* and *35S:MYB76* lines had an overall increase in total aliphatic glucosinolates with the most significant effects being on the longer of the short-chained glucosinolates, 5MSP, 6MTH and 6MSH (Table 3). Thus, all three MYB genes can specifically alter accumulation of aliphatic glucosinolates when over-expressed using a 35S promoter, and the compositional differences between the *35S:MYB* transgenes suggest that they are not operating via identical mechanisms.

**Table 3:** Average Seed Glucosinolate Content in transgenic *35S:MYB* lines.

Trait	Col-0		*MYB28*			*MYB29*			*MYB76*		
	N = 8		N = 11			N = 11			N = 13		
	Mean	SE	Mean	SE	Sig	Mean	SE	Sig	Mean	SE	Sig
3MTP	0.11	0.01	0.07	0.01	**	0.20	0.03		0.14	0.01	
3MSP	0.12	0.02	0.46	0.09	**	0.29	0.08		0.17	0.03	
3OHP	1.10	0.08	0.87	0.11		0.99	0.02		1.11	0.07	
3BZOP	1.56	0.09	1.59	0.11		1.83	0.05		1.75	0.06	
4MTB	13.63	1.01	18.91	1.13	*	17.51	1.07		16.87	0.63	
4MSB	1.44	0.17	6.26	1.61	**	2.71	0.64		2.15	0.29	
4OHB	1.88	0.17	1.45	0.13		1.57	0.11		2.17	0.14	
4BZOB	1.97	0.06	1.64	0.18		1.85	0.14		2.24	0.08	*
5MSP	0.10	0.01	0.71	0.20	**	0.26	0.06	**	0.18	0.01	**
6MTH	0.06	0.01	0.14	0.02	**	0.11	0.02	*	0.12	0.01	**
6MSH	0.07	0.00	0.18	0.08		0.13	0.02	*	0.10	0.01	**
7MTH	1.34	0.07	0.63	0.19	**	1.46	0.05		1.45	0.09	
7MSO	0.52	0.02	0.55	0.20		0.71	0.06	*	0.64	0.04	*
8MTO	2.25	0.14	0.42	0.15	**	2.03	0.11		2.14	0.14	
8MSO	4.06	0.17	2.44	0.67	*	4.60	0.13		4.73	0.29	
Total Aliphatic	30.97	1.57	37.99	3.41	*	37.42	1.85	*	37.01	1.20	*
I3M	0.80	0.06	0.75	0.09		0.66	0.06	*	0.72	0.05	
4OH-I3M	0.01	0.00	0.03	0.00	**	0.01	0.00		0.01	0.00	
Total Indole	1.05	0.06	0.95	0.11		0.86	0.07	*	0.99	0.05	
Isoleucine	0.74	0.08	1.53	0.23	**	1.11	0.13		0.99	0.10	

Mean shows the average glucosinolate content in nmol per mg of tissue using the abbreviations in Table S6. SE is the standard error of the mean for that line. This data represents using eight plants for Col-0, 11 plants containing the *35S:MYB28* transgene (five and six plants from two independent transgenic lines), eleven plants containing the *35S:MYB76* transgene (five and six plants each from two independent transgenic lines) and thirteen plants containing the *35S:MYB29* transgene (six and seven plants from two independent transgenic lines). Sig indicates the *P* value of the difference between Col-0 and transgenic lines containing the respective *35S:MYB* transgene as determined by ANOVA (Table S7). One asterisk represents a *P* value between 0.05 and 0.005 while two asterisks is a *P* value below 0.005. Cells with no asterisk represent non-significant *P* values, those greater than 0.05. N represents the total number of independent samples per genotypic class.

### Gene expression analysis in *35S:MYB* over-expression lines

The observation that over-expression of the three MYB genes resulted in elevated content of aliphatic glucosinolates led us to test if the transcript levels of genes in the biosynthetic pathway were concurrently elevated in the different genotypes. Affymetrix ATH1 genechip microarrays were used to measure transcript accumulation in wild-type and the two selected transgenic lines for each *35S:MYB* transgene. As for the glucosinolate analysis described above, an ANOVA analysis of transcript levels between the selected transgenic lines showed no significant difference, which allowed us to combine the mean of the two independent lines to test the effect of the introducing the MYB transgene rather than the effect of one single transgenic line versus wild-type.

Elevated accumulation of aliphatic glucosinolates might be expected to affect the entire sulphur metabolism of the plant due to the pull on the methionine pool. Consequently, we utilized a pathway ANOVA [24] approach to test the impact of the MYB over-expression on the major sulfur-utilization pathways, i.e. sulfate assimilation, cysteine production, methionine production, aliphatic glucosinolates, indole glucosinolates, homocysteine conversion and SAM production, as well as on the characterized transcription factors for indole and aliphatic glucosinolates (Table S8) . The predefined gene set for each individual pathway used in the pathway ANOVA are listed in Table S8.

**Figure 4:**
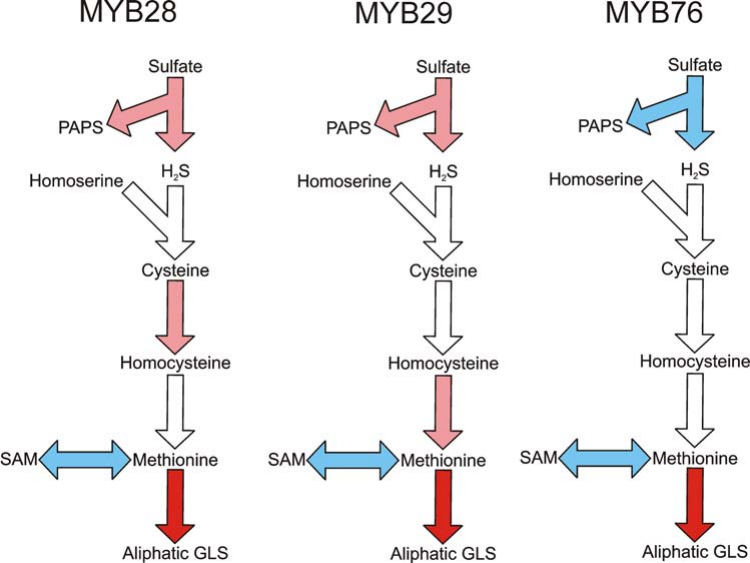
Expression of Sulfur Utilization Biosynthetic Pathways in *35S:MYB* lines. Nested ANOVAs were utilized on microarray data to test for altered expression of the major sulfur utilization biosynthetic pathways as described. The pathways linking one major metabolite to another with statistically significant altered expression are shown as colored arrows. Red shows that the *35S:MYB* lines led to increased transcript levels for the biosynthetic pathway in comparison to wild-type, while blue shows decreased transcript levels. Dark color represents a change of 50 percent or more while the lighter color shows a change of less than 50 percent. The genes used for each pathway are as listed in Table S8. MYB28 illustrates the comparison of transcript levels in *35S:MYB28* lines versus Col-0. MYB29 illustrates the comparison of transcript levels in *35S:MYB29* lines versus Col-0. MYB76 illustrates the comparison transcript levels in *35S:MYB76* lines versus Col-0.

The pathway ANOVA revealed that the primary effect of the three *35S:MYB* transgenes within these pathways is to induce the biosynthesis of aliphatic glucosinolates since this was the pathway showing the largest effect in both magnitude and statistical support (Figure 4 and Table S9). Another common effect of over-expression of the three MYB genes was to lower transcript levels for genes required to convert methionine into SAM. This could potentially increase the pool of methionine available for production of aliphatic glucosinolates (Figure 4 and Table S9). In addition, all three MYB genes altered transcript level for genes in the biosynthesis of PAPS (3′-phosphoadenosyl-5′-phosphosulfate) (sulfate assimilation genes, Table S8) which is the substrate required for the sulfotransferases catalyzing the final step of glucosinolate core synthesis (Figures 1 and 4). Interestingly, *MYB28* and *MYB29* induced the genes required for PAPS production whereas *MYB76* appeared to repress their transcript levels. As all plants for this experiment were grown side-by-side in a randomized design and all RNA extraction and microarray analysis conducted simultaneously, this difference is likely a valid biological observation rather than due to experimental error.

**Figure 5:**
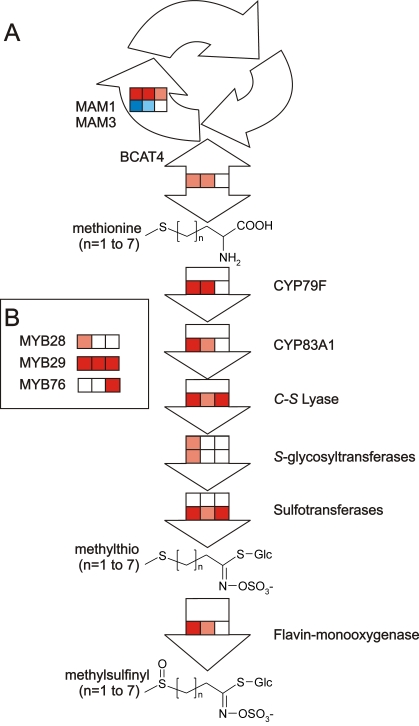
Altered Transcript Levels for Genes in the Biosynthetic Pathway of Aliphatic Glucosinolates in the *35S:MYB* lines. A. Altered transcript accumulation for the biosynthetic genes. B. Altered transcript accumulation for the MYB transcription factors by the different 35S transgenes. Nested ANOVAs were utilized on microarray data to test for altered transcript levels for biosynthetic genes in the aliphatic glucosinolate pathway. Each arrow represents a specific biosynthetic process with the transcript alteration for each of the different enzymes indicated as separate rows of boxes. From left to right, the boxes in each row illustrates the comparison of the transcript levels in, respectively, the *35S:MYB28*, *35S:MYB29* and *35S:MYB76* transgenes versus Col-0. The enzymes are listed in Table S10. Genes with a statistically significant altered transcript increase in the given *35S:MYB* line are shown as red while those with a decrease are in blue. Dark color represents a change of 50 percent or more while the lighter color shows a change of less than 50 percent.

Within the pathway ANOVAs, we next utilized F-tests to test if the transcripts for the individual genes in the biosynthesis of aliphatic glucosinolates were altered in comparison to wild-type Col-0. As expected, the individual *35S:MYB* lines led to elevated accumulation of the specific MYB gene that was over-expressed (Figure 5 and Table S10). However, even though the transcript levels of *MYB28* was significantly elevated, its modest increase of approximately 40% was in contrast to the more dramatic elevation of approximately 200 and 500% in transcript level of *MYB29* and *MYB76* within the *35S:MYB29* and *35S:MYB76* lines, respectively. The modest increase in *MYB28* expression is, however, sufficient to result in an increased accumulation of glucosinolates in the *35S:MYB28* lines (Tables 1 and 3). Further, *MYB29* transcript accumulated in response to over-expressing *MYB28* and *MYB76* (Figure 5 and Table S10) suggesting the presence of some interplay between the MYB genes.

Over-expression of *MYB28* and *MYB29* led to statistically significant increases in transcript levels for, respectively, 17 and 13 of the predicted and characterized genes in aliphatic glucosinolate biosynthesis or regulation (Table S10). In contrast to the overall induction of the aliphatic biosynthetic genes in the *35S:MYB* lines, a reduction in *MAM3* transcript levels was observed in *35S:MYB28* and *35S:MYB29* lines with the biggest difference occurring in *35S:MYB28* lines (Figure 5 and Table S10). The *35S:MYB76* lines upregulated relatively fewer transcripts of aliphatic biosynthetic genes as it showed altered transcript levels for only ten of the predicted and characterized genes in aliphatic biosynthesis and regulation (Figure 5 and Table S10). The ANOVA results obtained from pathways as well as the individual genes in aliphatic glucosinolate biosynthesis suggest that even though *MYB28*, *MYB29* and *MYB76* have significant functional overlap, they differ in their regulatory capacities or targets.

### Genome-wide transcript effects of *35S:MYB* over-expression lines

**Figure 6:**
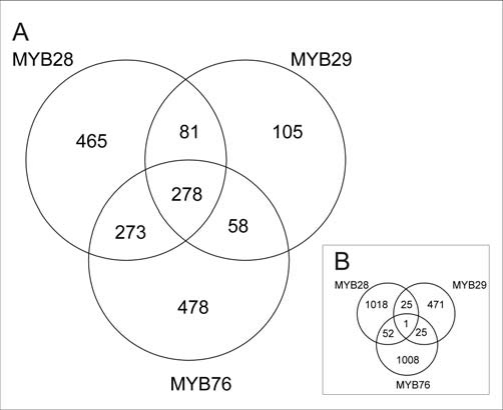
Overlap in Altered Gene Regulation Between the *35S:MYB* over-expressor lines. Each ring of the Venn diagram shows the number of genes whose transcript level was statistically significantly altered by the given *35S:MYB* transgene. Statistical significance was determined by individual gene ANOVAs using a FDR of 0.05. The bottom diagram shows the predicted number of genes in each intersection under the assumption that the MYB genes have independent regulatory functions.

To better assess the overlap of transcripts altered in *35S:MYB* lines, we conducted separate ANOVAs to test every transcript for a significant difference between wild-type and each selected *35S:MYB* line. Using a false discovery rate (FDR) of 0.05, data indicated that the *35S:MYB28* lines altered the accumulation of 1097 transcripts, the *35S:MYB29* lines 522 transcripts and the *35S:MYB76* lines 1087 transcripts (Figure 6 and Table S11). The effects were nearly equally divided between transcripts induced and those repressed (Table S11). In agreement with the hypothesis that all three *MYB* genes share regulatory targets such as aliphatic glucosinolates, there was a significant bias for overlap in transcripts regulated by all three *MYB* genes (*P*<0.001 by Chi-square, Figure 6). This overlap included approximately 53% of all transcripts regulated by *MYB29*. In contrast to the overlap, there are a number of genes altered by specific subsets of the MYBs further suggesting that they are not operating via a single mechanism (Figure 6). Decreasing the FDR rate to 0.10 did not alter the ratio of transcripts populating the regions of the Venn diagram suggesting that the indication of specificity amongst the factors is not merely a statistical artifact of the microarray analysis (data not shown). An analysis of GO annotations in all quadrants of the Venn diagram did not show any informative bias with regards to function of the genes whose transcripts were affected (data not shown).

Previous work has suggested a link between aliphatic glucosinolates and the accumulation of sinapate esters whereby the glucosinolate biosynthetic mutants altered sinapate accumulation and vice-versa [38], [39]. Interestingly, all three *35S:MYB* genotypes led to decreased transcript levels of *MYB4–At4g38620*, a MYB transcription factor which suppresses the accumulation of sinapate esters [40]. Accordingly, transcript levels of *SNG1*-*At2g22990*, the gene responsible for conversion of sinapoyl glucose to sinapoyl malate [41] (Table S11) are increased in all three genotypes. Curiously, however, *BRT1–At3g21560*, responsible for the conversion of sinapate to sinapoyl glucose [42], on the other hand, are down-regulated in all three. This suggests that the MYBs may be involved in the suggested cross-talk between sinapate and aliphatic glucosinolate metabolism.

### *myb28-1*, *myb29-1* and *-2* and *myb76-1* and *-2* T-DNA mutants display reduction in different aliphatic glucosinolates

**Figure 7:**
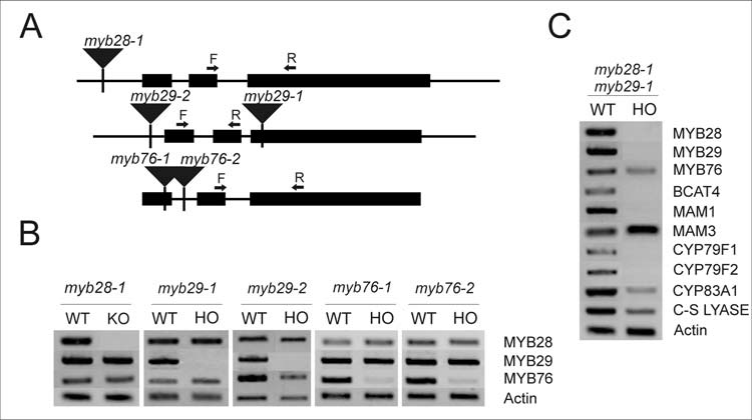
Characterization by RT-PCR of transcript levels in *myb28-1*, *myb29-1*, *myb29-2*, *myb76-1*, *myb76-2* and *myb28-1 myb29-1* mutants. A. Diagram of the *MYB28*, *MYB29* and *MYB76* genes with exons given as black boxes and 5′UTR, 3′UTR and introns given as black lines. The T-DNA insertion site in *myb28-1* is located in the 5′UTR, the T-DNA insertion sites in *myb29-1* and *myb29-2* are located in the third exon and 5′UTR, respectively, and the T-DNA insertions of *myb76-1* and *myb76-2* are located in the first exon and first intron, respectively. Arrows marked F and R show the approximate positions of the primers used for RT-PCR. B. Steady state foliar mRNA transcript levels of *MYB28*, *MYB29* and *MYB76* and various aliphatic biosynthetic genes in wild-type Col-0, *myb28-1*, *myb29-1, myb29-2*, *myb76-1* and *myb76-2* and *myb28-1 myb29-1* mutants as measured by RT-PCR in 23-25 days old plants. Each mutant is displayed with its corresponding wild-type. A PCR for actin was used as a loading control. Amplification was shown to be in the logarithmic phase. C. Steady state foliar mRNA transcript levels of *MYB28*, *MYB29* and *MYB76* and various aliphatic biosynthetic genes in wild-type Col-0 and the *myb28-1 myb29-1* mutant as measured by RT-PCR in 25 days old plants. A PCR for actin was used as a loading control. Amplification was shown to be in the logarithmic phase.

The 35S over-expression analysis provides a guide to a gene's potential activity but has the capacity to generate false-positive impressions. To validate that the MYB candidate genes play a role in biosynthesis of aliphatic glucosinolates *in planta*, loss-of-function alleles in *MYB28*, *MYB29* and *MYB76* were obtained [43]–[45] and the borders of the T-DNA insertions sequenced to validate the insertion sites (Figure 7A). The transcript levels for the MYB genes were measured in all five lines, *myb28-1*, *myb29-1*, *myb29-2, myb76-1* and *myb76-2*, to determine whether the T-DNA insertions resulted in a loss of transcript. RT-PCR was conducted on RNA purified from at least two independent wild-type plants and homozygous single-mutant plants and with at least two different cycle numbers to better quantify changes. The analysis revealed that *myb28-1, myb29-1* and *myb29-2* were indeed knockout mutants whereas *myb76-1* and *myb76-2* still had residual but much reduced *MYB76* transcript levels (Figure 7B). Given that the T-DNA insert is in an exon, it should be noted that *myb76-1* may be a functional knockout. The knockout or knockdown of one MYB transcription factor did not lead to changes in levels of any of the other MYB transcription factors. No apparent visual phenotype was observed in any of the single knockout mutants under the conditions tested. The impact on the transcript level for the individual biosynthetic genes in the knockouts was minimal with only a slight reduction in *BCAT4, MAM1* and *CYP79F1* transcripts in *myb29-1* and *myb29-2* and in *MAM1*, *CYP79F2* and *CYP83A1* transcripts in *myb28-1* (data not shown). It is possible that levels of aliphatic glucosinolates are sensitive to changes in aliphatic glucosinolate transcript accumulation that are not easily detectable using quantitative RT-PCR. This could be enhanced as the level of a transcripts is a single time-point measure while the level of aliphatic glucosinolates likely integrates over a length of time, thereby amplifying any change.

To minimize maternal effects and to show that any observed chemotype segregated with the T-DNA insertion, we genotyped germinating offspring from segregating heterozygous mutants and measured glucosinolates on the homozygous knockout and wild-type sibling progeny for each mutant. The combined mean from *myb29-1* and *myb29-2* and that of *myb76-1* and *myb76-2* are presented since ANOVA showed that there was no difference in the relative levels of glucosinolates between the different mutant alleles (Table S12). Leaves from the *myb29-1*, *myb29-2*, *myb76-1* and *myb76-2* mutants had significantly reduced levels of short-chained aliphatic glucosinolates content with no change in the amounts of the long-chained aliphatic glucosinolates (Table 4). In contrast, the *myb28-1* mutant showed a dramatic reduction in long-chained and a decrease in short-chained aliphatic glucosinolates (Table 4). These results indicate that *MYB29* and *MYB76* play a role for regulation of short-chained aliphatic glucosinolates, whereas *MYB28* plays a role in the control of both short- and long-chained aliphatic glucosinolates in leaves. The mutations in *MYB28*, *MYB29* and *MYB76* did not affect indole glucosinolate levels (Table 4).

**Table 4:** Foliar glucosinolate content in T-DNA insertion mutants of *MYB28*, *MYB29* and *MYB76*.

	*myb28-1*					*myb29*					*myb76*				
	WT		Homozygous			WT		Homozygous			WT		Homozygous		
	N = 6		N = 12			N = 24		N = 16			N = 6		N = 10		
	Mean	SE	Mean	SE	Sig	Mean	SE	Mean	SE	Sig	Mean	SE	Mean	SE	Sig
4MSB	140	37	65	20		90	9	69	11	**	60	6	40	4	*
4MTB	49	14	24	9		24	5	21	5	**	7	1	3	1	**
5MSP	11	2	7	2		8	1	5	1	**	7	1	5	0	
7MSH	8	2	2	1	**	6	0	8	1		5	0	5	0	
7MTH	5	2	1	0	**	3	1	5	2		1	0	1	0	
8MSO	38	11	5	1	**	26	2	37	0.1		20	2	20	2	
8MTO	1	0	0	0	**	2	0	3	6		1	0	2	0	
Total aliphatic	250	65	111	33		159	16	147	21		100	8	76	8	*
I3M	77	13	82	18		70	7	97	20		42	4	51	3	
4MO-I3M	4	1	5	1		5	0	5	1		5	0	5	0	
NMO-I3M	7	2	9	6		5	1	9	3		1	0	2	0	
Total indole	88	15	96	24		80	8	112	23		49	4	59	4	

Plants are derived from progeny of mutants heterozygous for the *myb28-1, myb29-1, myb29-2, myb76-1* and *myb76-2* alleles. Mean shows the average glucosinolate content in pmol per mg of fresh weight tissue using the abbreviations in Table S6. SE is the standard error of the mean for that line. This data represents two independent biological replicates, except for *myb76* which only has one replicate. The data for the two *myb29* alleles and the two *myb76* alleles were pooled as there was no significant difference in the glucosinolate phenotype between the different alleles (Table S12). Sig indicates the *P* value of the difference between Col-0 wild-type and the transgenic lines as determined by ANOVA (Table S12). One asterisk represents a *P* value between 0.05 and 0.005 while two asterisks is a *P* value below 0.005. Cells with no asterisk represent non-significant *P* values, those greater than 0.05. N represents the total number of independent samples per genotypic class.

The impact of the *myb28-1*, *myb29-1*, *myb29-2*, *myb76-1* and *myb76-2* insertions on the glucosinolate pool in seeds was measured on seeds derived from the plants on which glucosinolates in leaves had been measured. This showed that *myb76* only displayed a small decrease in 4MSB and 7MSH, indicating that this gene only plays a minor role in aliphatic glucosinolate regulation in seeds. However, both the levels of short and long-chained aliphatic glucosinolates were significantly reduced in homozygous *myb28-1* seeds (Table 5) leading to a substantial reduction in total amounts of aliphatic glucosinolates (Table 5). For *myb29*, a significant reduction was observed in the levels of the short-chained 4MTB, with no impact on long-chained aliphatic glucosinolates (Table 5). Curiously, 4BZOP levels were increased in both genotypes. The decrease of aliphatic glucosinolates in seeds further supports the observation that both *MYB28* and *MYB29* play a role in controlling the accumulation of aliphatic glucosinolates in leaves and seeds, but via different chain-length specificities. The level of indole glucosinolates was not affected in either line.

**Table 5:** Seed glucosinolate content in T-DNA insertion mutants of *MYB28*, *MYB29* and *MYB76*.

	myb28-1					myb29					myb76				
	WT		Homozygous			WT		Homozygous			WT		Homozygous		
	N = 7		N = 11			N = 25		N = 17			N = 6		N = 9		
	Mean	SE	Mean	SE	Sig	Mean	SE	Mean	SE	Sig	Mean	SE	Mean	SE	Sig
3BZOP	0.39	0.07	0.32	0.04	**	0.42	0.02	0.32	0.02	*	0.45	0.01	0.42	0.03	
4MTB	3.5	0.52	2.73	0.37	*	3.83	0.18	3.01	0.21	*	3.53	0.32	3.86	0.23	
4MSB	0.48	0.17	0.21	0.06	*	0.41	0.07	0.15	0.02		0.6	0.16	0.34	0.06	*
4OHB	0.27	0.04	0.3	0.04		0.31	0.02	0.34	0.03		0.32	0.05	0.34	0.03	
4BZOB	0.38	0.06	0.74	0.09	*	0.49	0.03	0.62	0.06	**	0.5	0.04	0.5	0.03	
5MTP	0.26	0.06	0.2	0.04	*	0.3	0.02	0.23	0.02		0.33	0.02	0.35	0.02	
7MTH	0.25	0.05	0	0	**	0.32	0.02	0.3	0.04		0.4	0.01	0.36	0.03	
7MSH	0.2	0.04	0.01	0.01	**	0.21	0.01	0.21	0.02		0.25	0.02	0.18	0.02	*
8MTO	0.38	0.06	0.01	0.01	**	0.55	0.04	0.54	0.07		0.59	0.05	0.56	0.03	
8MSO	0.87	0.14	0.04	0.03	**	1.07	0.06	0.93	0.1		1.2	0.05	1	0.08	
Total aliphatic	6.86	1.12	4.52	0.54	**	7.86	0.33	6.55	0.49	*	8.25	0.42	7.97	0.46	
I3M	0.18	0.05	0.28	0.04		0.21	0.01	0.22	0.02		0.25	0.02	0.22	0.02	

Seeds are derived from homozygous or wild-type progeny of a mutant heterozygous for the *myb28-1, myb29-1, myb29-2, myb76-1* and *myb76-2* alleles. Mean shows the average glucosinolate content in nmol/seed using the abbreviations in Table S6. SE is the standard error of the mean for that line. This data represents two independent biological replicates, except for *myb76* which only has one replicate. The data for the two *myb29* alleles were pooled as there was no significant difference in the glucosinolate phenotype between the different alleles (Table S13). Sig indicates the *P* value of the difference between Col-0 wild-type and the homozygous mutant lines as determined by ANOVA (Table S13). One asterisk represents a *P* value between 0.05 and 0.005 while two asterisks is a *P* value below 0.005. Cells with no asterisk represent non-significant *P* values, those greater than 0.05. N represents the total number of independent samples per genotypic class.

### A *myb28-1 myb29-1* double knockout mutant displays no detectable aliphatic glucosinolates and reduced transcript level of biosynthetic enzymes

**Figure 8:**
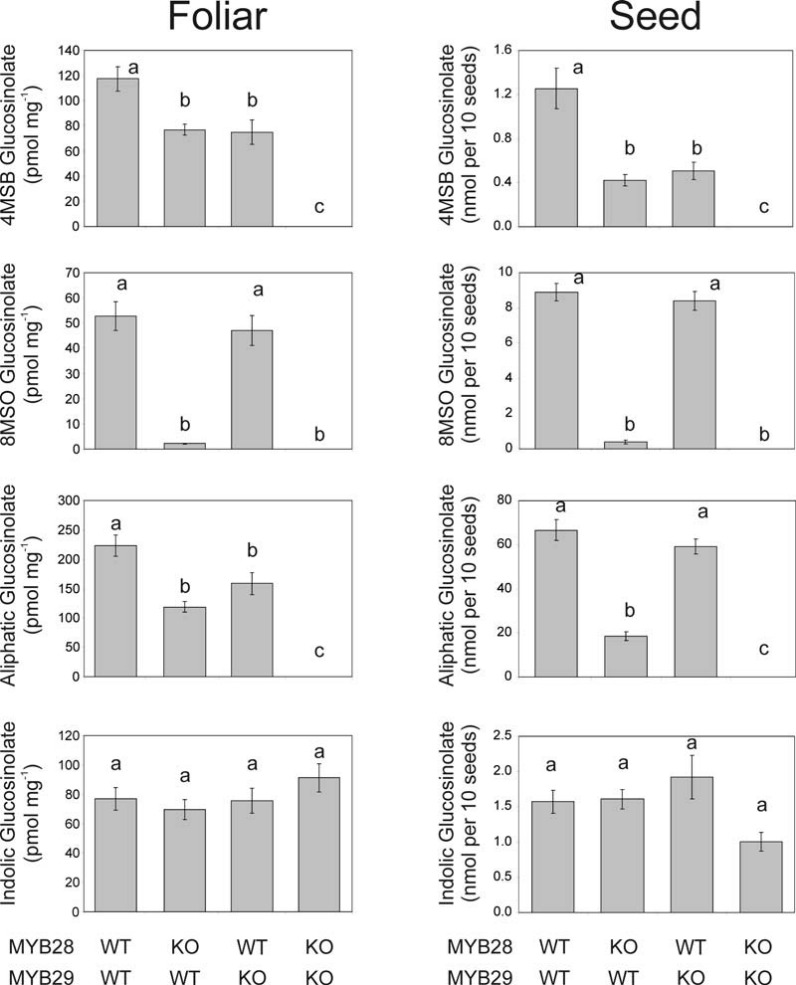
The effects of *myb28-1 myb29-1* Double Mutant on Glucosinolate Accumulation. Homozygous wild-type, homozygous single mutant or homozygous double mutant progeny were measured for foliar and seed glucosinolates by HPLC. 12 independent plants were separately measured per line for the four lines and the data analyzed via ANOVA (Table S14, S15, S16 and S17). Data for 4MSB, 8MSO, total aliphatic and total indolic glucosinolate content are shown. Genotypes with different letters show statistically different glucosinolate levels for the given glucosinolate.

To further investigate the roles of *MYB28* and *MYB29* on production of aliphatic glucosinolates, we crossed *myb28-1* and *myb29-1* to obtain a double knockout mutant. Homozygous double knockouts were obtained. Analysis of transcript accumulation within the homozygous double knockout in comparison to the WT Col-0 showed that transcripts of *BCAT4*, *MAM1*, *CYP79F1* and *CYP79F2* were undetectable in the double knockout leaves under the cycle numbers tested. Furthermore, a substantial reduction was observed in *CYP83A1* and *C-S-LYASE* transcripts (Figure 7C). Finally, the absence of *MYB28* and *MYB29* transcripts resulted in a small decrease in the transcript level of *MYB76* (Figure 7C). We were unable to detect aliphatic glucosinolates in either the leaves or seeds of the homozygous double *myb28-1 myb29-1* mutant (Figure 8). In comparison to the WT and homozygous single knockouts, this loss of glucosinolates showed a statistically significant epistatic interaction between MYB28 and MYB29. A merely additive interaction would have led to foliar total aliphatic glucosinolates having a level of 25% of wildtype in the double mutants (Figure 8 and Tables S14, S15, S16 and S17). This data confirm that in addition to having specific activities, *MYB28* and *MYB29* also have synergistic functionalities. Indole glucosinolate levels were not affected in the *myb28-1 myb29-1* double knockout mutant in comparison to the WT or homozygous single knockout lines (Table S14, S15, S16 and S17). The loss of aliphatic glucosinolates in the double knockout plants could not have been predicted by the chemotype of the single knockout mutants and as such reveals an emergent property of glucosinolate regulation. Additionally, this double knockout phenotype suggests that MYB76 requires a functional MYB28 or MYB29 to control aliphatic glucosinolates.

## Discussion

### Identification of aliphatic glucosinolate regulators

We have utilized diverse systems biology tools to identify *MYB28, MYB29* and *MYB76* as candidate regulators of aliphatic glucosinolates. This involved a combination of natural variation in both metabolite and transcript networks, phylogenetic analysis and bioinformatics of gene expression databases. When the three MYB genes were individually over-expressed in a wildtype Col-0 background, all lines accumulated more aliphatic glucosinolates in leaves and seeds than the corresponding wildtype (Tables 1–3). Microarray analysis showed that transcript levels for genes involved in biosynthesis of aliphatic glucosinolates in foliar tissues were concurrently up-regulated (Figure 5). This showed that all three MYB genes have the potential to up-regulate the accumulation of aliphatic glucosinolates via increasing the biosynthetic transcripts. Analysis of knockout mutants of *MYB28*, *MYB29* and *MYB76* further established a role of the three MYB genes in regulation of aliphatic glucosinolates in Col-0 since absent expression of the genes led to reduced contents of aliphatic glucosinolates, as evidenced by altered profiles in both leaves and seeds (Table 4–5). This is in contrast to the hypothesis proposed by Hirai *et al.* (2007) that *MYB29* has no major role in regulation of aliphatic glucosinolates, except in response to jasmonic acid.

The identification of these three MYBs within a single clade and their overlapping phenotypes in the *35S:MYB* over-expressor lines suggested that they may be a redundant gene family. However, analysis of the knockout lines at the metabolic level showed that these functions were not redundant as *MYB29* and *MYB76* controlled short-chained aliphatic glucosinolates and *MYB28* controlled both short- and long-chained aliphatic glucosinolates. The impact of *MYB28* on both chain lengths is likely mediated through an impact on *CYP79F* expression as the *MYB28*/*MYB29* double knockout does not impact MAM3 expression. Similar results were obtained with the *myb28-1* knockout by Hirai *et al.* (2007). The individual knockout mutants had only minimal effects on transcripts for the individual genes involved in biosynthesis of aliphatic glucosinolates (data not shown). In contrast, the *myb28-1 myb29-1* double knockout mutant dramatically diminished most transcripts for the biosynthetic genes and abolished the contents of all aliphatic glucosinolates (Figure 7C and Figure 8). This shows that this family of MYBs functions to regulate the aliphatic glucosinolate biosynthetic pathway in Col-0 and that they have evolved specific and overlapping functions that show complex interconnectivity.

### The *myb28-1 myb29-1* double knockout mutant reveals an emergent interaction of *MYB28* and *MYB29* on aliphatic glucosinolates in Col-0

The glucosinolate profiles of the single knockout mutants suggest that *MYB28* and *MYB29* play significant, but distinct roles in regulation of the biosynthetic genes for aliphatic glucosinolates as both lead to lower levels of specific aliphatic glucosinolates (Table 4 and 5). However, transcript levels were only minimally affected by mutations in the individual genes (data not shown). A *myb28-1 myb29-1* double knockout mutant showed that both genes apparently positively interact to control both transcript levels and metabolite accumulation for the majority of the pathway. The total level of aliphatic glucosinolates of the double knockout mutant were dramatically lower than either single knockout mutant in the leaves, and below the level of detection for all aliphatic glucosinolates in both leaves and seeds (Figure 8 and Table S14, S15, S16 and S17). In concordance, the transcripts of most characterized aliphatic biosynthetic genes were undetectable in the leaves of the double knockout mutant (Figure 7C). None of the phenotypes of the single mutants hinted at the striking phenotype of the double knockout mutant and, as such, the analysis of the latter identifies an emergent property of the glucosinolate regulation system not readily predictable from the phenotypes of the single knockout mutants.

The double knockout analysis points to an interplay between *MYB28* and *MYB29* whereby they interact to activate the aliphatic glucosinolate pathway. One source of possible *MYB* interplay was observed in the *35S:MYB* lines, where over-expression of *MYB28* and *MYB76* led to increased levels of *MYB29* transcript. This suggests a different role for *MYB29,* in which it integrates signals from *MYB28* and *MYB76* in regulating the aliphatic glucosinolates. However, this is not a strictly linear pathway where *MYB28* and *MYB76* would regulate *MYB29* to regulate the glucosinolates since *MYB29* transcript seems unchanged in the *myb28-1*, *myb76-1* and *myb76-2* mutants (Figure 7B). The observation that the *myb28-1 myb29-1* double knockout mutant altered transcripts more dramatically than either individual knockout suggests that *MYB28* has regulatory functionalities independent of *MYB29*. Future work is required to understand how the three *MYB* genes co-operatively stimulate the aliphatic glucosinolates.

### QTL co-localization

MYB28 co-localizes with one of the three major QTLs for aliphatic glucosinolate content and gene expression within the *Arabidopsis* Bay×Sha population (Figure 2 and [46]). Three lines of evidence strongly argue for *MYB28* being the basis for this QTL. First, *MYB28* has differential expression between Bay and Sha (Table S1). Second, altering *MYB28's* expression causes changes in aliphatic glucosinolate content and transcripts (Table 1, 4 and 5, Figure 5) . Third, *MYB28* is epistatic to both the *MAM* and *AOP* QTL [46] and has the ability to regulate expression of these genes. An additional QTL in the L*er*×Cvi population is likely caused by natural variation in *MYB76* expression [17]. Again, the gene has differential expression between L*er* and Cvi (http://www.weigelworld.org/resources/microarray/AtGenExpress/), this differential expression can lead to altered glucosinolate content and the QTL is epistatic to *MAM* and *AOP* whose genes are regulated by *MYB76.* Perlegen sequence data shows that there are polymorphisms between Bay/Sha for MYB28 and Ler/Cvi for MYB76 (Clark et al. 2007). However, none of these polymorphisms cause major effect polymorphisms. Additional crosses and verification are required to conclusively prove that MYB28 and MYB76 are indeed the genes underlying the total aliphatic QTLs in the two populations and to investigate what polymorphisms might be the underlying cause. Interestingly, the *AOP2* gene within the *AOP* QTL has recently been shown to lead to altered expression of *MYB28*, *MYB29* and *MYB76*, thus, establishing a molecular basis for their epistatic interactions. This sets up a highly interconnected regulatory network whereby variation in gene expression for the transcript regulators *MYB28*, *MYB76*, *MYB29* and *AOP2* can interact to generate a wide range of aliphatic glucosinolate content [16], [46]. As Col-0 does not have functional *AOP2*, it remains to be seen how this feed-forward loop actually functions within *Arabidopsis* [47]. The identification of these MYBs as the likely basis for two additional QTLs within *Arabidopsis* glucosinolate accumulation raises the possibility of using a similar systems biology approach to identify the complete molecular basis for quantitative variation in aliphatic glucosinolates.

### Regulation of substrate availability for aliphatic glucosinolates

An important factor in production of a given compound is the availability of precursor substrates. The similar level of total aliphatic glucosinolates (although with a different composition) in knockout mutants of either *MAM1* and *MAM3* [48] suggests that under normal conditions in Col-0, a predetermined amount of substrate is destined to go into the glucosinolate pathway. Over-expression of the *MYB* regulators allows more substrate to enter the glucosinolate pathway as evidenced by the observed increase of up to 110% in total aliphatic glucosinolate content (Table 1 and 2). This is reflected in the altered levels of transcripts for both the biosynthetic as well as for the substrate pathways (Figure 4), although we cannot conclude if the latter is a direct or indirect effect of the *MYB* over-expression. Compared to the variation in aliphatic glucosinolate content among the *Arabidopsis* accessions [49], the increase in aliphatic glucosinolate content modulated by the individual *MYB* genes can be regarded as rather modest. This suggests a putative restraint, when modifying a single gene, possibly due to a limitation in the substrate availability or in other components of the regulatory machinery. Interestingly, Gigolashvili *et al.* (2007) describe a line with a seven fold increase in 4MSB when over-expressing *MYB28*. However, this line shows a strong phenotype which could be due to a strong pull on the methionine pool. This suggests that when the production of aliphatic glucosinolates reaches a certain level due to, for instance, the manipulation of a single regulatory gene, plant growth is hampered by e.g. a shortage of methionine for protein biosynthesis.The alteration of expression levels of multiple genes within the natural accessions may allow for this bottleneck to be bypassed.

### *MAM3* might be differentially regulated from the other glucosinolate genes

The *MAM1* and *MAM3* genes have apparently arisen via a gene duplication and divergence in the past [50], [51]. Our data indicates that during this process, these two genes have obtained different regulatory patterns with *MAM1* acting similar to the other glucosinolate genes and *MAM3* being differently regulated. All three *35S:MYB* transgenes lead to an increase in *MAM1* and decrease in *MAM3* expression (Table S10) which suggests a reciprocal relationship. This is further substantiated by the observation that abolishment of one lead to upregulation of the other [48]. The *myb28-1 myb29-1* double knockout abolished most aliphatic glucosinolate related transcripts including *MAM1* but left *MAM3* unaffected or even slightly increased. The loss of long-chain aliphatic glucosinolates in this background is likely dependent upon the complete loss of expression for CYP79F1 and F2, removing the ability to convert the elongated homomethionines into glucosinolates. This suggests that the two genes have different regulatory networks rather than a relationship whereby the repression of one activates the other. Validating this hypothesis will require more analysis.

### Future Avenues

The rapidly increasing number of identified glucosinolate regulators, provides important tools to understand how the plant controls the production of these critical herbivore and pathogen defense compounds. Transcriptional analysis of double and triple knockout lines of the MYB genes may identify how they interact to control glucosinolate production as well as the metabolic networks required to provide substrates for the production.

The multiple duplications and divergences leading to the indole and aliphatic glucosinolate MYBs as well as in the genes which they regulate (e.g. *CYP79F1*/*F2* and *CYP79B2/B3*) provides means to address evolutionary questions, as these genes have evolved specific and overlapping functions that show complex interconnectivity over time. The glucosinolate biosynthetic genes and their regulators may be an excellent system for studies on how transcription factors have evolved simultaneously with their target genes to give rise to new biosynthetic pathways, such as the aliphatic and indole glucosinolates ones, with different regulation. Identification and validation of the regulatory role of *MYB28*, *MYB29* and *MYB76* as having the same general regulatory targets will greatly facilitate such evolutionary studies in the future.

## Materials and Methods

### QTL Mapping

The Bay-0×Sha population of 403 *A. thaliana* recombinant inbred lines [52] was used to map QTL controlling total aliphatic glucosinolate content. Seeds were imbibed and cold stratified at 4°C for three days to break dormancy. A single plant per line was grown in flats containing 96 cells per flat, and maintained under short day conditions in controlled environment growth chambers. Four flats contained a single replicate of each of the 403 lines. Two independent randomized replicate blocks were grown. At 35 days post germination, a fully-expanded mature leaf was harvested, digitally photographed and analyzed for total aliphatic glucosinolate content via HPLC. The Bay-0×Sha RIL population has previously been genotyped [25], [52]. The average total aliphatic glucosinolate content for the RILs was used for QTL mapping within Windows QTL Cartographer v2.5 [53]–[55]. Composite interval mapping (CIM) was implemented using Zmap (Model 6) with a 10 cM window and an interval mapping increment of 2 cM. Forward regression was used to identify five cofactors per quantitative trait. The declaration of statistically significant QTL is based on permutation derived empirical thresholds using 1,000 permutations for each trait mapped [56], [57]. Other QTL identified within this analysis will be presented elsewhere.

Further, 211 of these lines have been analyzed for variation in gene expression [22], which provided data that has been used to map eQTL controlling transcript levels for glucosinolate biosynthetic genes [17]. This allows comparison of QTL controlling metabolite accumulation with eQTL for transcript levels. The eQTLs for glucosinolate biosynthetic transcripts identified within the previous analysis were queried for the presence of an eQTL at the same position as found for the total aliphatic glucosinolate content QTL (Table S2).

### Plant cultivation

*Arabidopsis thaliana* ecotype *Colombia*, transgenics and mutants were grown in a growth chamber (HEMZ 20/240/S, Heraeus) at a photosynthetic flux of 100 µEi at 20°C and 70% relative humidity at a 16 h photoperiod in a soil:vermiculite (10∶1) mixture.

### Sequences

Different annotations of the size of mRNA and coding sequences of *MYB28* and *MYB29* are found in the databases. The *MYB28* mRNA sequence is found in two different versions in the NCBI database (http://www.ncbi.nlm.nih.gov/entrez/query.fcgidbNucleotide). One (NM_125535.) encodes a transcript of 1425 bp long and encodes for a protein of 367 amino acids. Another mRNA (NM_180910) is 1805 bp long and is predicted to encode a 288 amino acid protein. Similarly, two different *MYB29* mRNA sequences exist–one of 1595 bp (NM_120851) and one of 1292 bp (AF062872)–both are predicted to encode a 337 aa protein. The *MYB76* mRNA (NM_120852 , DQ446930 , AF175992 ) of 1017 bp is predicted to encode a protein of 339 aa. The two different coding sequences of *MYB28* were aligned with the annotated coding sequences of *MYB29* and *MYB76*. It was found that the 288 amino acid protein lacked the crucial R2 and most of the R3 DNA binding domain. Consequently, the 367 amino acid encoding region was regarded as the correct coding sequence and subsequently used in the clonings.

### Creation of transgenic plants

The over-expression constructs driven by the CaMV 35S promoter were created by cloning, respectively, *MYB28*, *MYB29* and *MYB76* coding regions from *Arabidopsis thaliana* ecotype *Columbia* cDNA into the pCAMBIA230035Su using the USER™ method as described [58]. Primer sequences used for the PCRs are summarized in Table S18. Binary plasmids were transferred to *Agrobacterium tumefaciens* strain C58 [59] and transformed into *Arabidopsis* plants according to the floral dip method [60]. Transgenic plants were selected on ½ MS with 50 mg L^−1^ kanamycin.

### DNA extraction and genotyping of T-DNA insertion mutants

Total DNA was extracted essentially as described in [61]. T-DNA insertions in *At5g61420* (line SALK_136312 = *myb28-1*), *At5g07690* (lines GABI_868E02 = *myb29-1* and SM.34316 = *myb29-2*) and *At5g07700* (lines SALK_096949 = *myb76-1* and SALK_055242 = *myb76-2*) were confirmed by PCR. *myb28-1* had a T-DNA insertion in the 5′UTR region of the gene, 182 bp upstream of the start codon. The T-DNAs of *myb29-1*and *myb29-2* are positioned in the third exon 730 bp upstream of the stop codon and in the 5′UTR 40 bp upstream the start codon, respectively. The T-DNAs of *myb76-1* and *myb76-2* are situated respectively, 99 bp downstream the ATG in the first exon and 194 downstream the ATG in the first intron. Two separate PCR reactions were carried out to identify the position of the insertion site and the zygosity of the plants. Forward and reverse primers were designed according to the SIGnAL T-DNA verification primer design tool (http://signal.salk.edu/tdnaprimers.2.html) for the SALK lines and with Primer3 (http://frodo.wi.mit.edu/cgi-bin/primer3/primer3_www.cgi) for the GABI line. The gene specific primers were used in combination with left border primers (LBa1 for SALK lines or 8409 LB for GABI lines and Spm32 for the SM-line) to verify the presence and orientation of the T-DNAs. Primer sequences used for genotyping are summarized in Table S18. Eppendorf HotMaster Taq DNA Polymerase (Hotmaster) (Eppendorf, AG, Hamburg, Germany) was used in a 20 µl reaction using 1 unit enzyme, 187.5 µM dNTP, buffer 1∶10 and 187.5 µM of each primer and DNA as template. The PCR program was as follows: Denaturation at 94°C for 3 min, 35 cycles of denaturation at 94°C for 30 s, annealing at 56°C for 30 s, extension at 65°C for 1.15 min and finally extension at 65°C for 3 min.

### *myb28-1 myb29-1* double mutant construction

To construct the double mutant, *myb28-1 myb29-1*, the homozygous *myb28-1* and *myb29-1* were crossed with each other. The F_1_ plant was self-fertilized and progeny in the F_2_ generation was genotyped by PCR (see above).

### RT-PCR on knockouts and wild-type

Leaves from homozygous *myb28-1* single knockout, homozygous *myb28-1 myb29-1* double knockout and *Arabidopsis* Columbia wild-type plants (all derived from a segregating *myb28-1 myb29-1* F2 plants) were harvested 25 days after germination. Leaves from plants homozygous for the absence or presence of the *myb29-1*, *myb29-2*, *myb76-1* and *myb76-2* allele were harvested 23 days after germination. RNA was extracted with Trizol reagent (Invitrogen, Carlsbad, CA) according to the manufacturer's instructions. The samples were DNAse treated with 2 units DNA free™ (Ambion, Cambridgeshire, Great Britain) according to the manufacturer's instructions. One µg of total RNA was reverse transcribed using the iScript cDNA Synthesis Kit (Biorad, Hercules, CA). The primers used for RT-PCR are listed in Table S18. PCR was performed with Eppendorf HotMaster Taq DNA Polymerase (Hotmaster) (Eppendorf, AG, Hamburg, Germany) in a 20 µl reaction using 1 unit enzyme, 187.5 µM dNTP, buffer 1∶10 and 187.5 µM of each primer and cDNA as template. The PCR program was as follows: Denaturation at 94°C for 3 min, 22–35 cycles of denaturation at 94°C for 30 s, annealing at 53–56°C for 30 s, extension at 65°C for 0.45–1.15 min and finally extension at 65°C for 3 min.

### Generation of plant material for glucosinolate analysis

T2 progeny of *35S:MYB28*, *35S:MYB29* and *35S:MYB76* lines was germinated on ½ MS with 50 mg/L^−1^ kanamycin. Resistant plants were used for the analysis along with *Arabidopsis thaliana* ecotype *Colombia* wild-type plants germinated on ½ MS. Plants were transferred to soil 10–12 days after germination. For the microarray experiment, transgenic plants were identified by PCR with primers for the *nptII* gene (Table S18). In the knockout experiments, seeds from a segregating heterozygous knockout mutants or heterozygous/heterozygous double knockout were germinated on soil and transferred to flats containing 48 or 96 cells per flat 10–12 days after germination. Leaves were harvested for glucosinolates analysis just before bolting 22–25 days after germination.

### Glucosinolate extraction and analysis

Glucosinolate extraction was performed as previously described [49]. For analysis of leaves, 30–100 mg leaves were used. For analysis of seeds, 5–10 mg or 10 seeds were used for the extraction. HPLC analysis was performed as previously described [33].

### LC-MS analysis

20 µl sample was injected by ASI-100 Automated Sample injector (Dionex, Denmark) and separated on a Zorbax SB-AQ RPC18 column (4.6 mm×250 mm, 5 um) (Agilent Technologies, USA) at a flow rate of 1 ml/min delivered by a P680 HPLC pump (Dionex). The program was as described previously for HPLC [33]. A STH585 column thermostate (Dionex) kept the column temperature at the set 25°C. The mobile phase was split using a T-piece and delivered 20% of the total flow (1 ml/min) to the mass spectrometer and 80% to an UV-detector equipped with a micro flow cell (UVD340S, Dionex) and the desulfoglucosinolates were detected at 229 nm. Mass spectrometry was carried out on a single quadrupole Thermo Finnigan Surveyor MSQ equipped with electrospray injection. The electrospray capillary voltage was set at 3 kV, the cone voltage at a constant 75 V and the temperature was 365°C. For ionization 50 µl/min of 250 µM NaCl was added to the flow (after split) using an AXP-MS high pressure pump (Dionex) and the desulfoglucosinolates were detected as [M+Na]^+^ adduct ions. Desulfoglucosinolates were identified according to *m/z* values and retention times and quantified by the A_229 nm_ and published response factors [37].

### Glucosinolate statistical analysis

The glucosinolate content was analyzed via ANOVA utilizing SAS proc glm. Each *35S:MYB* line was tested for altered glucosinolate content in an individual ANOVA against the wild-type Col-0 genotype. For each *35S:MYB* transgene, two independent transgenic lines were obtained and both transgenic lines grown in two independent experiments. Each transgenic line and Col-0 were measured from at least four individual plants within each experiment resulting in a minimal number of eight plants per line. The plants were measured for both seed and leaf glucosinolates as described above. For the ANOVA, the Genotype term tested for a difference between wild-type and the *35S:MYB* lines while the impact of the two independent lines per *35S:MYB* transgene were simultaneously tested by the nested factor, Transgene(Genotype). The above ANOVAs included factors to account for variation between the different experiments.

ANOVA was also utilized to test for the effect of the different knockout mutants. For the knockouts, segregating F2 progeny from heterozygous F1 individuals were screened to identify progeny homozygous for the presence of the T-DNA knockout mutant or homozygous for its absence. The homozygous lines were then measured for glucosinolate content in the seeds and leaves. For each T-DNA knockout mutant line, the experiment was conducted in two fully independent rounds with at least three plants per genotype per experiment for a minimal N of six. The Genotype term within the ANOVA was used to test for a difference between the progeny homozygous for the knockout mutant and those siblings missing the T-DNA. For *MYB29* and *MYB76*, there were two independent T-DNA mutant lines. Both lines were tested and the difference between the independent mutants tested as a nested factor using the factor Knockout(Genotype). This allowed us to test if the effects on glucosinolate accumulation were due to the presence of any T-DNA within *MYB29* and *MYB76* (Genotype) or if there was a difference between the separate T-DNA alleles [Knockout(Genotype)]. Sums-of Squares, F-values and P-values are presented (Table S12).

### Microarray Analysis of MYB Over-expression

Plants for the various genotypes were grown as previously described. At 25 days post germination, a fully-expanded mature leaf was harvested, weighed and analyzed for total aliphatic glucosinolate content via HPLC. The remaining plant material was collected, flash frozen and total RNA extracted via RNeasy columns (Qiagen, Valencia, CA, USA). Two independent plants were combined to provide sufficient starting material for a single RNA extraction. Two independent samples were obtained per transgenic line with two different transgenic lines per *35S:MYB* transgene, thus providing four-fold replication. Six wild-type Col-0 RNA samples were obtained. This provided a total of 18 independent microarrays. Labeled cRNA was prepared and hybridized, according to the manufacturer's guidelines (Affymetrix, Santa Clara, CA, USA), to whole genome Affymetrix ATH1 GeneChip microarrays, containing 22,746 *Arabidopsis* transcripts. The GeneChips were scanned with an Affymetrix GeneArray 2500 Scanner and data acquired via the Microarray Suite software MAS 5.0 at the Functional Genomics Laboratory (University of California Berkeley). RMA normalization was used to obtain gene expression levels for all data analyses [62].

### Microarray statistical analysis

The gene expression data was first analyzed via a network/biosynthetic pathway ANOVA approach utilizing the general linear model within SAS [24]. Sulfur utilization biosynthetic pathways were obtained from AraCyc v3.4 (http://www.arabidopsis.org/biocyc/) and modified to better organize the pathways based on metabolites of importance for glucosinolate synthesis (Table S8). Transcription factor networks for aliphatic and indole glucosinolates were added based on this research and previously published research [9], [13], [14]. Each selected, independent *35S:MYB* line was tested against the wild-type data in an independent ANOVA. For the ANOVA, the genes were nested factors within the higher order pathway. Additionally, the two independent lines per *35S:MYB* transgene were nested factors [Transgene(Genotype)] within the Genotype term (wild-type versus *35S:MYB*). This allowed us to test for effects due to the transgene versus the independent transgenic line. Each pathway was then tested within the model for a difference between the wild-type and *35S:MYB* lines using an F-test (Table S9). Additionally, we tested each aliphatic glucosinolate biosynthetic and transcription factor gene for altered transcript accumulation. These individual gene tests were also done within the confines of the model using an F-test to test for a mean separation between wild-type and the *35S:MYB* line (Table S10). All *P* values for these genes were significant after a FDR adjustment within the confines of this pathway ANOVA utilizing a pre-defined subset of genes meant to address a specific question about sulfur-utilization and glucosinolate biosynthetic pathways.

Next, the gene expression data was analyzed via individual gene ANOVA for each transcript. This was done by conducting ANOVA on each gene using the two independent transgenic lines per *35S:MYB* transgene as nested factors [Transgene(Genotype)] within the Genotype (WT versus *35S:MYB* transgene) effect. The ANOVA calculations were programmed into Microsoft Excel to obtain all appropriate Sums-of-Squares and to obtain the F values for the effect of the Genotype (wild-type versus *35S:MYB* transgene) and Transgene(Genotype) effects for each gene (Table S11). The nominal *P* values for both terms are presented as well as the *P* values for the Genotype (wild-type versus *35S:MYB* transgene) effect that are significant after a FDR adjustment to the 0.05 level (Table S11) [63].

## Supporting Information

Figure S1 Multiple Sequence Alignment Alignment of genes used for Phylogenetic Reconstruction.
(2.21 MB TIF)

Table S1 trans eQTL
(0.03 MB XLS)

Table S2 cis eQTL
(0.03 MB XLS)

Table S3 Gene Lists, tree
(0.03 MB XLS)

Table S4 Myb coexpression list
(0.42 MB XLS)

Table S5 35SMYB Foliar Stats
(0.03 MB XLS)

Table S6 Abbreviations
(0.09 MB XLS)

Table S7 35SMYB Seed Stats
(0.03 MB XLS)

Table S8 Pathway Lists
(0.03 MB XLS)

Table S9 Pathway Results
(0.02 MB XLS)

Table S10 Aliphatic GLS Array
(0.03 MB XLS)

Table S11 Microarray data on 35S:MYB overexpressors
(8.79 MB XLS)

Table S12 Foliar MYB KO Stats
(0.03 MB XLS)

Table S13 Seed MYB KO Stat
(0.03 MB XLS)

Table S14 Foliar DoubleKO Stats
(0.02 MB XLS)

Table S15 Foliar means doubleKO
(0.02 MB XLS)

Table S16 Seed DoubleKO stats
(0.02 MB XLS)

Table S17 Seed means doubleKO
(0.02 MB XLS)

Table S18 Primers
(0.02 MB XLS)
