# Supplementary figures and images for: A Systems Biology Approach Identifies a R2R3 MYB Gene Subfamily with Distinct and Overlapping Functions in Regulation of Aliphatic Glucosinolates
Source: PLoS One. 2007 Dec 19;2(12):e1322. doi: 10.1371/journal.pone.0001322 (PMC2147653; doi:10.1371/journal.pone.0001322)

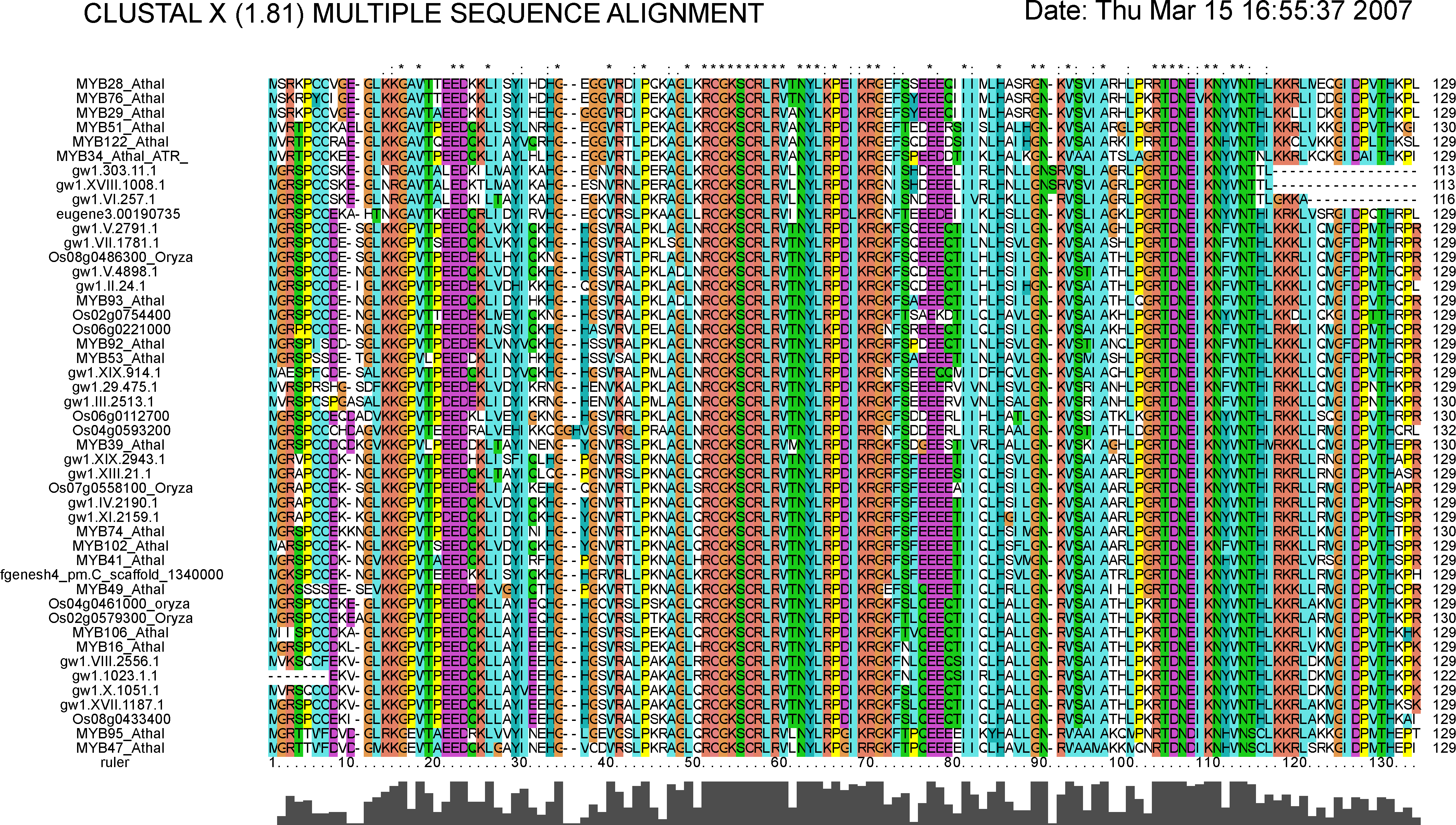

Supplement: Figure S1 — Multiple Sequence Alignment Alignment of genes used for Phylogenetic Reconstruction. (2.21 MB TIF) [file pone.0001322.s001.tif]
